# Author Correction: Single-cell RNA sequencing technologies and bioinformatics pipelines

**DOI:** 10.1038/s12276-021-00615-w

**Published:** 2021-05-27

**Authors:** Byungjin Hwang, Ji Hyun Lee, Duhee Bang

**Affiliations:** 1 Department of Chemistry, Yonsei University, Seoul, Korea; 2 Department of Clinical Pharmacology and Therapeutics, College of Medicine, Kyung Hee University, Seoul, Korea; 3 Kyung Hee Medical Science Research Institute, Kyung Hee University, Seoul, Korea

**Keywords:** Biological techniques, Genetics

Correction to: *Experimental & Molecular Medicine*

10.1038/s12276-018-0071-8 published online 07 August 2018

After online publication of this article, the authors noticed an error in the missing reference section for the Figure 3c.

The correct statement of this article should have read as below.

Additional reference for Figure 3c should be added in the Figure 3c legend and reference section as below and original reference number through 53–103 needs to be re-numbered as 54–104 since new reference^53^ is added: **c** A schematic example illustrates the difference between the RPKM and TPM measures^53^. TPM is efficient for measuring relative abundance because total normalized reads are constant across different cells.

The authors apologize for any inconvenience caused.

The original article has been corrected.
